# Comments on Ahmad, I.M. et al. Healthcare Workers Occupationally Exposed to Ionizing Radiation Exhibit Altered Levels of Inflammatory Cytokines and Redox Parameters. *Antioxidants*, 2019, *8*, 12

**DOI:** 10.3390/antiox8020042

**Published:** 2019-02-15

**Authors:** Joseph J. Bevelacqua, Seyed Mohammad Javad Mortazavi

**Affiliations:** 1 Bevelacqua Resources, 343 Adair Drive, Richland, WA 99352, USA; bevelresou@aol.com; 2 Medical Physics Department, Shiraz University of Medical Sciences, Shiraz 7134845794, Iran; 3 Diagnostic Imaging Department, Fox Chase Cancer Center, 333 Cottman Avenue, Philadelphia, PA 19111, USA

This commentary is regarding the paper recently published by Ahmad et al. (Healthcare Workers Occupationally Exposed to Ionizing Radiation Exhibit Altered Levels of Inflammatory Cytokines and Redox Parameters, doi: 10.3390/antiox8010012.). The authors measured levels of superoxide (O_2_•^−^) in whole blood and plasma levels of cytokines, oxidative DNA damage, extracellular superoxide dismutase (EcSOD) activity, and reduced/oxidized glutathione ratio (GSH/GSSG) in 20 radiation workers and 40 control subjects. Their study showed that the levels of O_2_•^−^ were significantly higher in the radiation workers compared to those of controls. Moreover, the radiation workers had a significant increase in the levels of interleukin (IL)-6, IL-1α, and macrophage inflammatory protein (MIP)-1α compared to controls. This paper has some major shortcomings. The first shortcoming is due to a large number of confounding factors (e.g., exposure to relatively high levels of radiofrequency radiation emitted from sources such as mobile phones, WiFi routers, and electronic equipment) thatare entirely ignored in this study. Substantial evidence now indicates the oxidative effects of low intensity radiofrequency radiation. Another major shortcoming comes from the great heterogeneity of the samples. Moreover, in the case of radiation therapy, a variety of radiation types with widely varying relative biological effectiveness values are utilized. In summary, sample sizes, mixing worker types, dose ranges, and worker characteristics limits the usefulness of this paper. Although the paper authored by Ahmad et al. addresses a very important topic, itcould have been improved if the issues noted in this commentary were addressed.

We read with interest the paper recently published by Ahmad et al. [1]. The authors measured levels of superoxide (O_2_•^−^) in whole blood and plasma levels of cytokines, oxidative DNA damage, extracellular superoxide dismutase (EcSOD) activity, and reduced/oxidized glutathione ratio (GSH/GSSG) in 20 radiation workers and 40control subjects. They reported that the levels of O_2_•^−^ were significantly higher in the radiation workers compared to those of controls. Moreover, the radiation workers had a significant increase in the levels of interleukin (IL)-6, IL-1α, and macrophage inflammatory protein (MIP)-1α compared to controls. Given this consideration, the authors concluded "*In view of the importance to improve understanding of the long-term health effects in workers occupationally exposed to radiation, Low [sic] dose radiation effect studies have to be one of the main research priority [sic]. Thus, follow-up evaluation of occupational health status, [sic] should be considered an integral part of quality assurance programs*" [1].

While commendable for its attempt to address a challenging topic [1], this paper has some major shortcomings. The first shortcoming is due to a large number of confounding factors (e.g., exposure to relatively high levels of radiofrequency radiation emitted from sources such as mobile phones, WiFi routers, and electronic equipment) thatare entirely ignored in this study. Yakymenko et al. have recently reported that 93 out of 100 peer-reviewed papers included in their study on the oxidative effects of low intensity radiofrequency radiation confirmed that these radiations induce oxidative effects in biological systems [2].

It is worth noting that alcohol consumption is among the few confounding factors that has been addressed and, interestingly, the consumption is higher in radiation workers! Another major shortcoming comes from the great heterogeneity of the samples. Among 20 cases,12 were involved in“conventional radiography”,four in“interventional radiography”, and four in “Computed Tomography (CT)”.It should be noted thatconventional radiography shows a hormetic response, and four radiation workers cannot ideally represent interventional or CT workers. Similar issues occur for personnel involved in therapy procedures.

In the case of radiation therapy, a variety of radiation types with widely varying relative biological effectiveness values are utilized. For example, Ahmad et al. [1] do not specifically consider the differences produced by beams of gamma-rays, protons, heavy ions, or neutrons that are used in various therapy applications [3,4,5]. The influence of different energy beams is also not evaluated. There are also differences in the doses received by various workers (e.g., radiation control technicians, radiologists, medical physicists, nurses, and other support personnel) during various therapy procedures [3,4,5].

In summary, mixing worker types, dose ranges, and worker characteristics limits the usefulness of this paper. The sample sizes are also limited and do not permit drawing definitive conclusions. Failing to consider or provide a justification for limiting the inclusion of confounding factors is an additional omission. The work of Ahmad et al. [1] addresses an important topic, but it could have been improved if the issues noted in this correspondence were addressed.

## Abbreviations

O_2_•^−^	superoxide
EcSOD	extracellular superoxide dismutase
GSH/GSSG	reduced/oxidized glutathione ratio
